# Myopia Prevalence Among 6–17 Years Students in Rural Areas of Seven Provinces of China

**DOI:** 10.3390/jcm15093261

**Published:** 2026-04-24

**Authors:** Xue Li, Huayu Zhang, Xiao Fang, Xiaodi Wu, Qian Gan, Yingying Huang, Qian Zhang, Hao Chen, Jinhua Bao

**Affiliations:** 1 National Engineering Research Center of Ophthalmology and Optometry, Eye Hospital, Wenzhou Medical University, Wenzhou 325027, China; lixue2016@eye.ac.cn (X.L.);; 2 Key Laboratory of Public Nutrition and Health, National Health Commission of the People’s Republic of China, National Institute for Nutrition and Health, Chinese Center for Disease Control and Prevention, Beijing 102206, China

**Keywords:** myopia, children and adolescents, ethnic, region

## Abstract

**Background/Objectives**: Estimate the prevalence of myopia among children aged 6–17 years in county and rural areas across seven geographically diverse provinces of China, and identify demographic, behavioral, and geographic factors associated with myopia, with particular focus on urban–rural and ethnic differences. **Methods**: A multi-stage stratified cluster sampling design was employed. Seven provinces were randomly selected, one from each of seven geographical regions of China (Southeast, North, Central, South, Southwest, Northwest, and Northeast). In each province, one rural county was randomly chosen. Within each county, one urban survey site (county town) and one rural survey site (village) were selected. From each site, one primary school and one junior high school were included. In each school, approximately 20 ± 2 students per grade (grades 1–9) were recruited. Uncorrected visual acuity and non-cycloplegic autorefraction were measured. Multivariable generalized linear mixed models (GLMM) with random intercepts at the class level were used to identify factors associated with myopia, accounting for the cluster sampling design. **Results**: The overall myopia prevalence was 42.9% (urban 49.6%, rural 36.0%). In the multivariable GLMM, educational stage was the strongest risk factor (grades 7–9 vs. 1–3: OR = 5.54). A significant district × ethnicity interaction was found only for Mongolian children: rural residence was strongly protective (OR = 0.19) compared to Han (OR = 0.65), and the ethnic advantage disappeared in county towns. Only 14.2% of myopic students had adequate correction. **Conclusions**: In conclusion, myopia is highly prevalent and severely under-corrected in rural China. Educational pressure is the main risk factor, and the rural protective effect is strongest in Mongolians but erodes with urbanization. Urgent public health actions, including vision screening, affordable spectacles, and lifestyle preservation, are needed to address this growing burden.

## 1. Introduction

In recent years, the global prevalence of myopia has risen alarmingly, marked by a trend toward earlier onset and higher incidence [1,2]. It is estimated that by 2050, half of the global population may have myopia, and 10% may have high myopia [1]. As the prevalence rises, visual impairment caused by myopia-related fundus pathologies such as myopic maculopathy and the associated socioeconomic burden also increase significantly [3]. The development of myopia is driven by a complex interplay of genetic predisposition and environmental factors [4]. In the Chinese context, population-based studies have further identified key environmental and socioeconomic determinants, including the level of economic development, educational pressures, seasonal variations in daylight exposure, and nutritional factors [5,6,7,8,9]. He. et al. quantified a pronounced disparity in myopia prevalence between major urban centers and rural populations, thereby anchoring the disease within the discourse on rapid socioeconomic transition and behavioral change [10,11].

However, the epidemiological understanding of myopia in China has been constrained by methodological limitations inherent in single-region or paired-comparison designs. These approaches lack the geographic granularity and demographic breadth necessary to disentangle the specific contributions of urbanization from the profound contextual heterogeneity—encompassing disparities in economic development, educational infrastructure, ethnic distributions, and cultural practices—that exists across the nation. This critical methodological gap has resulted in an incomplete and potentially biased national picture, leaving the variation of myopia’s urban–rural gradient across diverse regional and ethnic settings poorly quantified and underexplored.

To address this critical gap, the present cross-sectional study employs a multi-site, geographically diverse representative framework. By investigating county towns and rural schools across seven geographically stratified provinces, this design affords a unique opportunity to conduct a comparative analysis of myopia prevalence and visual impairment. It enables an examination of the consistency of urban–rural disparities while specifically exploring the modulating effects of regional and ethnic determinants. This investigation within the predominant county-level demographic aims to generate a nuanced, evidence-based foundation essential for formulating targeted and equitable public health strategies for myopia prevention.

## 2. Materials and Methods

### 2.1. Design and Subjects

This study adhered to the tenets of the Declaration of Helsinki and was approved by the Ethics Committee of the National Institute for Nutrition and Health (Approval No. 2021-018), and Eye Hospital, Wenzhou Medical University (Approval No. 2021-201-K-175). Written informed consent was obtained from all participating children and their parents or guardians prior to the study. The Strengthening the Reporting of Observational Studies in Epidemiology (STROBE) guidelines for cross-sectional studies were followed. A completed STROBE checklist with corresponding page numbers is available in Table S1.

**Figure 1:**
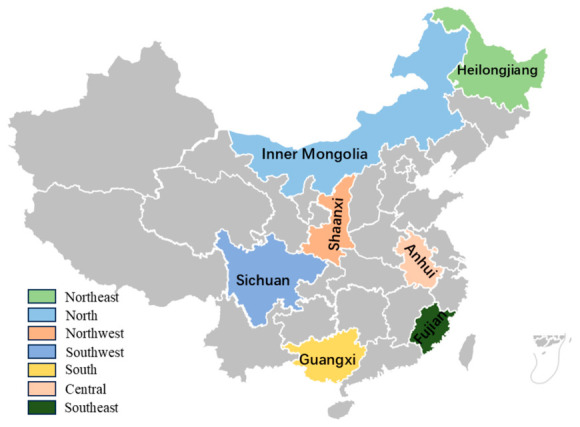
Distribution of surveyed provinces.

This cross-sectional study utilized a multi-stage stratified cluster sampling within the National Nutrition Improvement Program for Rural Compulsory Education Students. Seven key surveillance counties were strategically selected to ensure geographical diversity (spanning Northeast, North, Northwest, Southwest, South, Southeast, and Central China, Figure 1). The selected provinces were Heilongjiang (Northeast), Inner Mongolia (North), Shaanxi (Northwest), Sichuan (Southwest), Guangxi (South), Fujian (Southeast), and Anhui (Central). Within each province, one rural county was randomly selected as the survey site. In each county, one county town (urban) and one rural village were selected. In each of these two locations, one primary school (grades 1–6) and one junior high school (grades 7–9) were chosen. Within each school, one class per grade (grades 1–9) was randomly selected. All students in the selected class were invited to participate, with a target of approximately 20 students per class (balanced by gender when possible). This yielded a planned sample of 2520 students (7 provinces × 1 county × 2 locations × 2 schools × 9 grades × 20 students = 2520).

Participants were eligible for inclusion if they: (1) were enrolled in the selected primary or junior high schools; (2) completed both uncorrected visual acuity and non-cycloplegic autorefraction examinations; and (3) provided written informed consent from a parent or legal guardian, along with child assent. Participants were excluded if they: (1) used orthokeratology (OK) lenses; or contact lenses; (2) had any of the following ocular conditions: strabismus, nystagmus, ocular trauma, or known pathological myopia complications (e.g., myopic maculopathy, retinal detachment) that could affect refraction; or (3) had a history of ocular surgery.

### 2.2. Visual Acuity and Refractive Examination

Unaided visual acuity and habitual corrected visual acuity were measured using a liquid crystal digital (LED) visual chart (WSVC-1000, QDSGVision, Wenzhou, China) compliant with the Chinese national standard (GB11533). The testing distance was 5 m, left eye followed by right eye, recorded using the 5-point recording [12].

Non-cycloplegic autorefraction was performed using an autorefractor (KR-800, Topcon, Tokyo, Japan). The spherical equivalent (SE) was calculated as sphere + ½ cylinder. Five measurements were taken for each eye, and the average value was used. Measurements with poor reliability or high variability were repeated. Non-cycloplegic autorefractors can overestimate myopia, this study applies a composite definition—integrating both visual acuity and refractive error, to mitigate this potential overestimation. Specifically, per the International Myopia Institute (IMI) consensus definitions, myopia is defined as the presence of both unaided visual acuity (VA) < 5.0 and a spherical equivalent (SE) ≤ −0.50 diopters (D) in either eye [13,14,15]. Low myopia is defined as −6.00 D < SE ≤ −0.50 D with VA < 5.0; high myopia is defined as SE ≤ −6.00 D with VA < 5.0.

### 2.3. Behavioral Assessment

Behavioral data were collected by trained staff through face-to-face interviews with each child (or parent/guardian). Participants reported weekday and weekend hours of daytime outdoor activity and near-work (homework + screen time). Average daily time was calculated as (weekday × 5 + weekend × 2)/7.

### 2.4. Sample Size

Based on an expected myopia prevalence of 50%, a 3% margin of error, 95% confidence, a design effect of 2.0 (to account for cluster sampling), and a 10% non-response rate, the minimum required sample size was calculated as 2371 students.

### 2.5. Statistical Analysis

To analyze the effect of academic progression on myopia, the variable “educational stage” was defined as follows: 1–3 grade, 4–6 grade, and 7–9 grade. Because age and educational stage were highly correlated (Pearson’s r = 0.91), we orthogonalized age by regressing it on educational stage and saved the unstandardized residual (RES_1). This residual represents the component of age that is uncorrelated with educational stage, allowing simultaneous estimation of both variables without multicollinearity.

Statistical analysis was performed using SPSS 27.0. Measurement data with normal distribution are presented as mean ± standard deviation; categorical data are presented as frequency and percentage. Group comparisons were made using *t*-tests or ANOVA for normally distributed data, rank-sum tests for non-normally distributed data, and chi-square tests for proportions.

To account for the multistage cluster sampling design (students nested within classes), we used generalized linear mixed models (GLMM) with a random intercept at the class level. The binary outcome (myopia, yes/no) was modeled with a binomial distribution and a logit link function. Fixed effects included: educational stage (categorical), province, ethnicity, gender, RES_1 (continuous), district (county town vs. rural), daily outdoor time (continuous), daily near-work time (continuous), and the district × ethnicity interaction term. Odds ratios (ORs) and 95% confidence intervals (CIs) were calculated from the fixed effects. All statistical tests were two tailed, with a significance threshold set at *p* < 0.05.

## 3. Results

A total of 2532 students from the seven provinces were initially recruited and completed visual acuity and refractive examinations. Following the application of inclusion and exclusion criteria, 13 participants were subsequently excluded due to specific ocular conditions (2 strabismus, 2 nystagmus,1 ocular trauma) or the 8 use of contact lenses with unspecified correction details. Consequently, 2519 participants were included in the final analysis.

The analyzed cohort had a mean age of 10.8 ± 2.7 years (range: 6–18 years). The sample comprised 1307 males (51.9%) and 1212 females (48.1%). In terms of residence, 1271 participants (50.5%) were from county towns, and 1248 (49.5%) were from rural areas. Ethnically, the participants identified as Han (n = 1592, 63.2%), Qiang (n = 344, 13.7%), Zhuang (n = 276, 11.0%), Mongolian (n = 253, 10.0%), or from other ethnic groups (n = 54, 2.1%). The detailed demographic characteristics stratified by region are presented in Table 1.

Significant differences in mean age were observed across the seven provinces (One-way ANOVA, *p* < 0.001). Post hoc tests (Bonferroni) indicated that students from Heilongjiang (11.89 ± 2.63 years) and Inner Mongolia (11.45 ± 2.65 years) were significantly older than those from other provinces (all *p* < 0.05).

### 3.1. Prevalence of Myopia

The overall prevalence of myopia among the surveyed students was 42.9%, with a notable sex disparity (46.0% in females vs. 39.9% in males, Table 2). A significant urban–rural gradient was observed, with students residing in county towns exhibiting a higher prevalence compared to their rural students (49.6% vs. 36.1%; Table 2). Myopia prevalence demonstrated a strong positive association with educational stage, increasing significantly with higher grade levels (χ^2^ = 429.472, *p* < 0.001). The prevalence was 30.0% in primary school students and 68.3% in junior high school students. Marked geographical variations in myopia prevalence were identified across the seven provinces (χ^2^ = 62.553, *p* < 0.001). The prevalence ranged from 29.6% in Fujian to 56.8% in Heilongjiang Province, with the following descending order: Guangxi (49.4%), Anhui (42.2%), Shaanxi (41.5%), Inner Mongolia (41.3%), and Sichuan (39.6%). The distribution of myopia severity (low myopia vs. high myopia) across the provinces is further detailed in Table 2.

### 3.2. Behavioral Characteristics

Among the 2519 participants, 2450 (97.3%) had complete data on daily outdoor time and near-work time.

The mean daily outdoor time was 0.86 ± 0.82 h in non-myopic students and 0.99 ± 0.82 h in myopic students (mean difference = 0.13 h, 95% CI: 0.06–0.19; *p* < 0.001). The mean daily near-work time was 2.30 ± 2.00 h in non-myopic students and 3.22 ± 1.98 h in myopic students (mean difference = 0.80, 95% CI: 0.76–1.07; *p* < 0.001

### 3.3. Multivariable Analysis of Factors Associated with Myopia

The analysis revealed that educational stage was the strongest associated factor. Compared to grades 1–3, students in grades 4–6 had 3.3 times higher odds of myopia (OR = 3.32, 95% CI: 2.43–4.53, *p* < 0.001), and those in grades 7–9 had 5.5 times higher odds (OR = 5.54, 95% CI: 3.07–9.99, *p* < 0.001). Gender was also associated: female students had higher myopia odds than males (OR for male = 0.66, 95% CI: 0.55–0.79, *p* < 0.001), equivalent to 52% higher odds in females.

The age residual (within-grade age deviation) was a significant risk factor (OR = 1.16 per year, 95% CI: 1.03–1.30, *p* = 0.012), indicating that older students within the same grade have higher myopia risk. Daily near-work time showed a modest positive association (OR = 1.10 per hour increment, 95% CI: 1.03–1.17, *p* = 0.005). Daily outdoor time was not significantly associated (OR = 0.97, 95% CI: 0.84–1.12, *p* = 0.684).

Significant provincial differences were observed. Compared to Anhui province (central China), Fujian province (Southeast China) had lower myopia odds (OR = 0.47, 95% CI: 0.30–0.74, *p* = 0.001). No other province differed significantly from Anhui.

A significant district × ethnicity interaction was found (*p* = 0.004). Rural Mongolian students had 71% lower myopia odds than urban Han students (OR = 0.29, 95% CI: 0.13–0.68; interaction *p* = 0.004). No other ethnic group showed a significant interaction with district. Detailed results are presented in Table 3.

## 4. Discussion

This cross-sectional study investigated the prevalence and associated factors of myopia among students aged 6–18 from rural areas across seven provinces of China. The principal findings revealed that while the overall myopia prevalence (42.9%) was lower than the national average (which includes major cities), significant disparities were evident across geographic, and county-rural strata. Moreover, the rate of adequate optical correction was critically low at 14.2% (Table S2).

The overall myopia prevalence was 42.9% was lower than the 2018 national average of 53.6% [16]. This difference is likely attributable to our sampling strategy, which intentionally excluded major urban centers, where prevalence is typically higher [8]. A notable geographic disparity was observed that Fujian Province (Southeast China) exhibited the lowest prevalence (29.6%). Located near 25° N, this subtropical region features abundant sunshine and a mild climate, conductive to sustained outdoor activity, that is a well-established protective factor against myopia [17,18]. The mild seasonal variation in this subtropical area, characterized by shorter, less severe winters, may facilitate year-round outdoor exposure, thereby sustaining the protective effect against myopia. In contrast, students from Heilongjiang Province had a higher risk. The prolonged northern winter not only limits outdoor activity due to cold temperatures and reduced daylight hours, but also entails a marked seasonal reduction in both the duration and intensity of sunlight exposure. This likely diminishes retinal dopamine signaling, which is known to suppress axial elongation [19]. Consistent with this hypothesis, previous studies have demonstrated that myopia progression and axial elongation are significantly slower in summer than in winter [20,21]. Moreover, population-level analyses have established a negative association between sunshine duration and myopia prevalence in Chinese children and adolescents [22]. These hypotheses about climate influenced lifestyles are speculative, as we did not directly measure daylight exposure or seasonal behavioral changes. Other region-specific factors, such as dietary patterns, may also contribute to this disparity.

Consistent with national trends of a narrowing urban–rural myopia [2], our data show that rural myopia rates are rising and approaching urban levels. A significant district × ethnicity interaction was found only for Mongolian children, that rural residence was protective in all groups but much stronger in Mongolian (OR = 0.19) than in Han (OR = 0.65, Table S3). In county towns, no ethnic difference existed; while in rural areas, Mongolian children had lower odds than Han (OR = 0.22, Table S3). Previous studies in Inner Mongolia have consistently shown that Mongolian populations have lower myopia prevalence than Han populations, both in adults [23] and in school-aged children [24]. Moreover, nationwide multi-ethnic surveys have found that several minority groups (Tibetan, Uyghur, Yi, Yugur) also exhibit lower myopia rates than Han [25]. These studies also reported that rural or suburban residence is associated with lower myopia risk [23,24,25]. However, none of these studies specifically tested the interaction between ethnicity and urban–rural residence. Our study is the first to demonstrate that the protective effect of rural living is significantly stronger in Mongolian children than in Han children, and that this ethnic advantage disappears in county towns. This finding suggests that the lower myopia risk in Mongolian populations may not an intrinsic ethnic trait, but rather reflects the preservation of traditional pastoral lifestyles, which is characterized by more outdoor activities, less academic pressure, and unique dietary habits, and this characteristic gradually disappears with urbanization.

Furthermore, our findings strongly implicate educational stage as a principal factor associated with myopia. After adjusting for covariates, including age and near-work time, a pronounced dose-response relationship persisted, students in grades 4–6 had 3.3 times higher odds of myopia (OR = 3.32, 95% CI: 2.43–4.53), and those in grades 7–9 had 5.5 times higher odds (OR = 5.54, 95% CI: 3.07–9.99), compared to those in grades 1–3. respectively, compared to those in grades 1–3. In addition, each additional hour of daily near-work time was associated with a 10% increase in the odds of myopia (OR = 1.10, 95% CI: 1.03–1.17, *p* = 0.005). This observation is strongly supported by a nationwide study employing a regression discontinuity design, which concluded that each additional year of school education, rather than age itself, is a key independent risk factor for myopic refractive shift [26]. The IMI Risk Factors for Myopia report further confirmed that a causal link between increased years of education and more myopia by Mendelian randomization [27]. Moreover, our age residual analysis revealed that younger students within the same grade had higher myopia risk, suggesting that delayed school entry and reduced early academic pressure may be protective.

Female sex was independently associated with higher myopia odds (OR for male = 0.67, *p* < 0.001), with no interactions by district or grade. This consistent female predominance is supported by meta analyses [28] and may be explained by earlier pubertal development in girls [29], which triggers earlier axial elongation and myopia progression.

Among myopic students, only 14.2% had adequate optical correction (Table S2). Correction rates were significantly lower in rural areas (10.4% vs. urban 16.8%), in upper primary grades (3.9% vs. lower primary 18.0%), and in provinces such as Sichuan (6.3%) and Guangxi (7.6%). No gender difference was observed. This low correction rate is consistent with previous reports from China (e.g., 60% uncorrected in Shantou schools [30]; only 18.9% spectacle owned in rural China [31]). Uncorrected myopia impairs academic performance and quality of life, and may accelerate progression [32,33]. We recommend strengthening school-based vision screening, expanding optometry services in underserved regions, and providing subsidized spectacles for low-income families.

This study has limitations. Firstly, we defined myopia using non-cycloplegic autorefraction combined with uncorrected visual acuity (UCVA) rather than cycloplegic refraction which is the gold standard. Non-cycloplegic refraction overestimates myopia prevalence, especially in younger children, due to residual accommodation. However, combining UCVA with non-cycloplegic refraction significantly reduces this overestimation compared to refraction alone, and for school-aged children (≥6 years) this combination is considered sufficient for screening purposes [34,35]. Given the practical constraints of large-scale epidemiological surveys in resource-limited settings, our approach represents a pragmatic balance between accuracy and feasibility. Secondly, our sample was drawn from specific low-income rural areas where myopia surveillance is underdeveloped and healthcare resources are scarce. While the findings may not be generalizable to urban or high-income populations where myopia prevalence has plateaued 2, they provide valuable evidence for underserved rural regions that are often neglected in myopia research. Additionally, ethnicity and province-level factors are inherently correlated in our dataset (e.g., Han vs. other ethnic groups show different myopia risks across regions). We used generalized linear mixed models (GLMM) to partially account for clustering and collinearity, but residual multicollinearity may still affect the stability of estimates. Thirdly, this cross-sectional study precludes causal inference; all reported associations are correlational. Despite adjusting for several confounders (near work, outdoor time, age, etc.), residual confounding from unmeasured factors (e.g., diet, genetic markers, educational pressure) cannot be entirely excluded. While we followed standardized protocols to minimize this bias, residual selection bias remains possible.

## 5. Conclusions

In conclusion, myopia is highly prevalent and severely under-corrected in rural China. Educational pressure is the main driver, while the rural protective effect is strongest in Mongolians but erodes with urbanization. Urgent public health actions, including vision screening, affordable spectacles, and lifestyle preservation, are needed to address this growing burden.

## Figures and Tables

**Table 1:** General characteristics of the study population among seven provinces.

Variable	Total	Anhui	Fujian	Shaanxi	Sichuan	Guangxi	Inner Mongolia	Heilongjiang	*p*
No. of students	2519	358	358	359	361	344	380	359	
Age, years	10.80 ± 2.70	10.19 ± 2.68	10.37 ± 2.62	10.39 ± 2.60	10.53 ± 2.64	10.74 ± 2.65	11.45 ± 2.65	11.89 ± 2.63	<0.001
Sex									0.76
Boys, n (%)	1307 (51.9)	191 (53.4)	180 (50.3)	179 (49.9)	200 (55.4)	180 (52.3)	192 (50.5)	185 (51.5)	
Girls, n (%)	1212 (48.1)	167 (46.6)	178 (49.7)	180 (50.1)	161 (44.6)	164 (47.7)	188 (49.5)	174 (48.5)	
Educational stage									1.00
Grade 1–3	833 (33.1)	119 (33.2)	119 (33.2)	120 (33.4)	119 (33.0)	114 (33.1)	124 (32.6)	118 (32.9)	
Grade 4–6	841 (33.4)	119 (33.2)	120 (33.5)	120 (33.4)	121 (33.5)	115 (33.4)	128 (33.7)	118 (32.9)	
Grade 7–9	845 (33.5)	120 (33.5)	119 (33.2)	119 (33.1)	121 (33.5)	115 (33.4)	128 (33.7)	123 (34.3)	
Ethnic									<0.001
Han, n (%)	1592 (63.2)	358 (100.0)	357 (99.7)	359 (100.0)	10 (2.8)	40 (11.6)	113 (29.7)	355 (98.9)	
Mongolia, n (%)	253 (10.0)	-	-	-	-	-	253 (66.6)	-	
Qiang, n (%)	344 (13.7)	-	-	-	344 (95.3)	-	-	-	
Zhuang, n (%)	276 (11.0)	-	-	-	-	276 (80.2)	-	-	
Others, n (%)	54 (2.1)	-	1 (0.3)	-	9 (1.9)	28 (8.1)	14 (3.7)	4 (1.1)	
District									1.00
County town, n (%)	1271 (50.5)	179 (50.0)	179 (50.0)	180 (50.1)	182 (50.4)	174 (50.6)	194 (51.1)	183 (51.0)	
Rural area, n (%)	1248 (49.5)	179 (50.0)	179 (50.0)	179 (49.9)	179 (49.6)	170 (49.4)	186 (48.9)	176 (49.0)	

**Table 2:** Distributional characteristics of myopia in children and adolescents in this study.

Variable	No. of Students	Myopia		Low Myopia		High Myopia	
		Yes (1080) n (%)	Univariate Analysis	Yes (1026) n (%)	Univariate Analysis	Yes (54) n (%)	Univariate Analysis
Sex							
Males, n (%)	1307	522 (39.9)	χ^2^ = 9.556, *p* = 0.002	492 (37.6)	χ^2^ = 10.732, *p* = 0.001	30 (2.3)	χ^2^ = 0.298, *p* = 0.585
Females, n (%)	1212	558 (46.0)		534 (44.1)		24 (2.0)	
Educational stage							
Grade 1–3	833	152 (18.2)	χ^2^ = 429.472, *p* < 0.001	151 (18.1)	χ^2^ = 358.671, *p* < 0.001	1 (0.1)	χ^2^ = 44.716, *p* < 0.001
Grade 4–6	841	351 (41.7)		338 (40.2)		13 (1.5)	
Grade 7–9	845	577 (68.3)		537 (63.6)		40 (4.7)	
Ethnic							
Han, n (%)	1592	704 (44.2)	χ^2^ = 19.881, *p* < 0.001	670 (42.1)	χ2 = 16.510, *p* = 0.002	34 (2.1)	χ^2^ = 7.253, *p =* 0.123
Mongolia, n (%)	253	81 (32.0)		80 (31.6)		1 (0.4)	
Qiang, n (%)	344	135 (39.2)		126 (36.6)		9 (2.6)	
Zhuang, n (%)	276	136 (49.3)		129 (46.7)		7 (2.5)	
Others, n (%)	54	24 (44.4)		21 (38.9)		3 (5.6)	
District							
County town, n (%)	1271	630 (49.6)	χ^2^ = 46.923, *p* < 0.001	630 (49.6)	χ^2^ = 34.402, *p* < 0.001	7 (2.5)	χ^2^ = 12.313, *p* < 0.001
Rural area, n (%)	1248	450 (36.1)		450 (36.1)		3 (5.6)	
Provinces							
Anhui	358	151 (42.2)	χ^2^ = 62.553, *p* < 0.001	144 (40.2)	χ^2^ = 51.905, *p* < 0.001	7 (2.0)	χ^2^ = 9.403, *p* = 0.152
Fujian	358	106 (29.6)		104 (29.1)		2 (0.6)	
Shaanxi	359	149 (41.5)		141 (39.3)		8 (2.2)	
Sichuan	361	143 (39.6)		133 (36.8)		10 (2.8)	
Guangxi	344	170 (49.4)		162 (47.1)		8 (2.3)	
Inner Mongolia	380	157 (41.3)		151 (39.7)		6 (1.6)	
Heilongjiang	359	204 (56.8)		191 (53.2)		13 (3.6)	

**Table 3:** Multivariable generalized linear mixed model analysis of factors associated with myopia among students in seven provinces (N = 2450).

	OR (95%CI)	*p*-Value
Age residual (RES_1)	1.16 (1.03–1.30)	0.012
Daily near-work time	1.10 (1.03–1.17)	0.005
Daily outdoor time	0.97 (0.84–1.12)	0.684
Gender		
Female	Ref	
Male	0.66 (0.55–0.79)	<0.001
Educational stage		
Grade 1–3	Ref	
Grade 4–6	3.32 (2.43–4.52)	<0.001
Grade 7–9	5.54 (3.07–9.99)	<0.001
District		
County town	Ref	Ref
Rural	0.65 (0.48–0.89)	0.007
Ethnic		
Han	Ref	
Mongolian	0.76 (0.38–1.52)	0.438
Qiang	0.65 (0.20–2.07)	0.463
Zhuang	0.96 (0.44–2.11)	0.923
Others	0.72 (0.28–1.83)	0.493
Province		
Anhui	Ref	
Shaanxi	0.86 (0.55–1.35)	0.513
Fujian	0.47 (0.30–0.74)	0.001
Guangxi	1.35 (0.63–2.92)	0.444
Sichuan	1.69 (0.53–5.41)	0.373
Inner Mongolia	1.30 (0.65–2.57)	0.457
Heilongjiang	1.35 (0.81–2.25)	0.246
District × ethnicity		
county town × Han	Ref	
Rural × Mongolian	0.29 (0.13–0.68)	0.004
Other interactions	-	>0.05

Ref: Reference group (comparator). OR = 1.00 by definition; other groups are interpreted relative to this group.

## Data Availability

Dataset available on request from the authors—The raw data supporting the conclusions of this article will be made available by the authors on request.

## Supplementary Materials

The following supporting information can be downloaded at: https://www.mdpi.com/article/10.3390/jcm15093261/s1, Table S1: STROBE Statement—Checklist of items that should be included in reports of cross-sectional studies; Table S2: Proportion of myopic students with adequate optical correction by subgroup (N = 1080); Table S3: Simple effect analysis of the district × ethnicity interaction on myopia.

## Abbreviations

The following abbreviations are used in this manuscript:

IMI	International Myopia Institute
SE	spherical equivalent
VA	visual acuity
