# Supplementary material for: Myopia Prevalence Among 6–17 Years Students in Rural Areas of Seven Provinces of China
Source: J Clin Med. 2026 Apr 24;15(9):3261. doi: 10.3390/jcm15093261 (PMC13163749; doi:10.3390/jcm15093261)
Supplement: Supplementary file 1 [file jcm-15-03261-s001.zip › jcm-4219178-supplementary-Table S1.pdf]

Table S1. STROBE Statement—Checklist of items that should be included in reports of ***cross-sectional studies***

	Item No	Recommendation	Page No
**Title and abstract**	1	(*a*) Indicate the study's design with a commonly used term in the title or the abstract	P1
		(*b*) Provide in the abstract an informative and balanced summary of what was done and what was found	P1
**Introduction**			
Background/rationale	2	Explain the scientific background and rationale for the investigation being reported	P2
Objectives	3	State specific objectives, including any prespecified hypotheses	P2
**Methods**			
Study design	4	Present key elements of study design early in the paper	P3
Setting	5	Describe the setting, locations, and relevant dates, including periods of recruitment, exposure, follow-up, and data collection	P3
Participants	6	(*a*) Give the eligibility criteria, and the sources and methods of selection of participants	P3
Variables	7	Clearly define all outcomes, exposures, predictors, potential confounders, and effect modifiers. Give diagnostic criteria, if applicable	P4
Data sources/ measurement	8*	For each variable of interest, give sources of data and details of methods of assessment (measurement). Describe comparability of assessment methods if there is more than one group	*P4P5*
Bias	9	Describe any efforts to address potential sources of bias	P4P11P12
Study size	10	Explain how the study size was arrived at	P5
Quantitative variables	11	Explain how quantitative variables were handled in the analyses. If applicable, describe which groupings were chosen and why	P5
Statistical methods	12	(*a*) Describe all statistical methods, including those used to control for confounding	P5
		(*b*) Describe any methods used to examine subgroups and interactions	P5
		(*c*) Explain how missing data were addressed	P5
		(*d*) If applicable, describe analytical methods taking account of sampling strategy	
		(*e*) Describe any sensitivity analyses	
**Results**			
Participants	13*	(a) Report numbers of individuals at each stage of study—eg numbers potentially eligible, examined for eligibility, confirmed eligible, included in the study, completing follow-up, and analysed	P7
		(b) Give reasons for non-participation at each stage	P7

		(c) Consider use of a flow diagram	Not used; described in text
Descriptive data	14*	(a) Give characteristics of study participants (eg demographic, clinical, social) and information on exposures and potential confounders	Table 1
		(b) Indicate number of participants with missing data for each variable of interest	P5P7
Outcome data	15*	Report numbers of outcome events or summary measures	P5P7
Main results	16	(*a*) Give unadjusted estimates and, if applicable, confounder-adjusted estimates and their precision (eg, 95% confidence interval). Make clear which confounders were adjusted for and why they were included	Table 2 (crude), Table 3 (adjusted ORs with 95% CI)
		(*b*) Report category boundaries when continuous variables were categorized	P4P5
		(*c*) If relevant, consider translating estimates of relative risk into absolute risk for a meaningful time period	Not applicable
Other analyses	17	Report other analyses done—eg analyses of subgroups and interactions, and sensitivity analyses	eTable 1 (correction rates); eTable 2 (simple effects of interaction)
**Discussion**			
Key results	18	Summarise key results with reference to study objectives	P9
Limitations	19	Discuss limitations of the study, taking into account sources of potential bias or imprecision. Discuss both direction and magnitude of any potential bias	P9P10
Interpretation	20	Give a cautious overall interpretation of results considering objectives, limitations, multiplicity of analyses, results from similar studies, and other relevant evidence	P9-11
Generalisability	21	Discuss the generalisability (external validity) of the study results	P10
**Other information**			
Funding	22	Give the source of funding and the role of the funders for the present study and, if applicable, for the original study on which the present article is based	P11

*Give information separately for exposed and unexposed groups.

Note: An Explanation and Elaboration article discusses each checklist item and gives methodological background and published examples of transparent reporting. The STROBE checklist is best used in conjunction with this article (freely available on the Web sites of PLoS Medicine at http://www.plosmedicine.org/, Annals of Internal Medicine at http://www.annals.org/, and Epidemiology at http://www.epidem.com/). Information on the STROBE Initiative is available at www.strobe-statement.org.
