# Supplementary material for: Myopia Prevalence Among 6–17 Years Students in Rural Areas of Seven Provinces of China
Source: J Clin Med. 2026 Apr 24;15(9):3261. doi: 10.3390/jcm15093261 (PMC13163749; doi:10.3390/jcm15093261)
Supplement: Supplementary file 1 [file jcm-15-03261-s001.zip › jcm-4219178-supplementary-Tables S2 and S3.pdf]

Supplementary

Table S2. Proportion of myopic students with adequate optical correction by subgroup (N=1080)

Subgroup	Category	Corrected (n, %)	Uncorrected/Under-corrected (n, %)	χ^2	p-value
District	County town	106 (16.8)	524 (83.2)	8.790	0.003
	Rural	47 (10.4)	403 (89.6)		
Educational stage	grades 1-3	104 (18.0)	473 (82.0)	21.176	<0.001
	grades 4-6	6 (3.9)	146 (96.1)		
	grades 7-9	43 (12.3)	308 (87.7)		
Gender	Female	86 (15.4)	472 (84.6)	1.473	0.225
	Male	67 (12.8)	455 (87.2)		
Province	Anhui	21 (13.9)	130 (86.1)	23.798	0.001
	Shaanxi	33 (22.1)	116 (77.9)		
	Sichuan	9 (6.3)	134 (93.7)		
	Guangxi	13 (7.6)	157 (92.4)		
	Inner Mongolia	23 (14.6)	134 (85.4)		

Subgroup	Category	Corrected (n, %)	Uncorrected/Under-corrected (n, %)	χ^2	p-value
	Heilongjiang	36 (17.6)	168 (82.4)		
	Fujian	18 (17.0)	88 (83.0)		
Total		153 (14.2)	927 (85.8)		

*For myopic children, adequate optical correction was defined as a corrected visual acuity better than 4.8.

Table S3. Simple effect analysis of the district × ethnicity interaction on myopia

Comparison	OR (95% CI)	p-value
**Rural vs. county town within each ethnic group**		
Han	0.65 (0.48–0.89)	0.007
Mongolian	0.19 (0.10–0.37)	<0.001
**Mongolian vs. Han within each district**		
County town	0.76 (0.38–1.52)	0.438
Rural	0.22 (0.12–0.42)	<0.001

*
